# The DUNDRUM Quartet: validation of structured professional judgement instruments DUNDRUM-3 assessment of programme completion and DUNDRUM-4 assessment of recovery in forensic mental health services

**DOI:** 10.1186/1756-0500-4-229

**Published:** 2011-07-03

**Authors:** Sarah O'Dwyer, Mary Davoren, Zareena Abidin, Elaine Doyle, Kim McDonnell, Harry G Kennedy

**Affiliations:** 1 National Forensic Mental Health Service, Central Mental Hospital, Dundrum, Dublin 14, Ireland; 2 Department of Psychiatry, Trinity College, Dublin, Ireland

**Keywords:** Forensic, mental health, rehabilitation, recovery, risk, needs assessment

## Abstract

**Background:**

Moving a forensic mental health patient from one level of therapeutic security to a lower level or to the community is influenced by more than risk assessment and risk management. We set out to construct and validate structured professional judgement instruments for consistency and transparency in decision making

**Methods:**

Two instruments were developed, the seven-item DUNDRUM-3 programme completion instrument and the six item DUNDRUM-4 recovery instrument. These were assessed for all 95 forensic patients at Ireland's only forensic mental health hospital.

**Results:**

The two instruments had good internal consistency (Cronbach's alpha 0.911 and 0.887). Scores distinguished those allowed no leave or accompanied leave from those with unaccompanied leave (ANOVA F = 38.1 and 50.3 respectively, p < 0.001). Scores also distinguished those in acute/high security units from those in medium or in low secure/pre-discharge units. Each individual item distinguished these levels of need significantly. The DUNDRUM-3 and DUNDRUM-4 correlated moderately with measures of dynamic risk and with the CANFOR staff rated unmet need (Spearman r = 0.5, p < 0.001).

**Conclusions:**

The DUNDRUM-3 programme completion items distinguished significantly between levels of therapeutic security while the DUNDRUM-4 recovery items consistently distinguished those given unaccompanied leave outside the hospital and those in the lowest levels of therapeutic security. This data forms the basis for a prospective study of outcomes now underway.

## Background

We have recently validated structured professional judgement instruments for the triage of mentally disordered offenders and those like them to aid in the assessment of need for appropriate levels of therapeutic security [1,2], and to help prioritise the most urgent cases for admission. We have shown that the factors taken into account when deciding whether a patient should be admitted to an open admission unit, a locked low secure unit or a forensic high/medium secure unit are different from risk assessment instruments. It follows that the factors taken into account when deciding to move a patient from one level of therapeutic security to another, when deciding to allow leave from hospital or to discharge to the community may also be more complex than risk assessment. Since the factors taken into account when assessing the need for therapeutic security are essentially static the factors influencing decisions regarding progress to less secure places are likely to be different and inherently sensitive to change.

Once admitted, patients progress from admission/high secure to medium secure and on to pre-discharge low secure and community placements [3]. These placements correspond to a pathway through the secure treatment and rehabilitation service towards recovery, and also correspond to a stratified risk management system [4]. Levels of therapeutic security in a forensic hospital also correspond to measures of risk of harm to others and to self, symptom severity and global function [5-7]. Others have shown that changes in symptom score and function [8] and changes dynamic risk measures [9] relate to discharge. However decisions regarding risk management such as the level of leave permitted to individual patients are still largely made on the basis of implicit knowledge i.e. unstructured professional judgment rather than evidence based approaches [10]. The development of the structured professional judgement instruments described in this paper arose from the concern that when making the case for progress from high to medium, from medium to low secure placements or to the community, whether conditionally discharged or absolutely discharged, risk assessment did not fully describe the clinical and related factors taken into account. The evidence presented by clinicians to Mental Health Review Boards or Tribunals and other decision making authorities regarding leave outside the hospital or moves down the ladder of security and into the community is often influenced by factors such as stability, insight, rapport and working alliance, use of leave, assessments of dynamic risk factors and victim sensitivities. When writing reports to such decision making bodies, the emphasis was commonly on physical and mental health, substance misuse, problem behaviours, self care and activities of daily living, education, occupation and creative activities and family and social networks. While these overlap with the content of risk assessment instruments such as the HCR-20 [9] and the S-RAMM [11] or START [12] they are sufficiently different in content to justify drafting and testing structured professional judgement instruments specific for the function of assessing and communicating readiness for onward movement to less secure therapeutic placements.

The DUNDRUM Quartet [2] is a set of four structured professional judgement instruments modelled on the HCR-20 in that each consists of operationally defined ratings for domains relevant to the decision in hand. The DUNDRUM-3 Programme Completion Items and DUNDRUM-4 Recovery Items (see Additional File 1) are grounded in theory concerning motivation, the cycle of change, engagement and addressing issues relevant to future avoidance of relapse and problem behaviours. The rating scheme for each item is designed so that those rated '4' are unlikely to be ready for a move from their current secure setting. Those rated '3' should be ready for a move from a high secure to a medium secure setting; those rated '2' should be ready for a move from medium to low security; those rated '1' may be ready for a move to an open or community placement, though the availability of a high level of community support, structure and supervision, mandated if necessary by legally binding conditional discharge with a power of recall, may be a part of such a decision. Finally, those rated '0' may be ready for an absolute legal discharge though this should be an individual decision in all cases.

In this cross-sectional study we have tested the psychometric properties of these scales. We have also examined the extent to which they correspond to proxy measures of outcome. This study is a preliminary step in a prospective validation of the predictive power of these scales.

We hypothesized (i) that these items would form two psychometrically distinct scales. We hypothesized (ii) that the scales would have acceptable psychometric properties including internal consistency and inter-rater reliability; (iii) they were expected to correlate to some extent with measures of mental state and global function and (iv) with each other. We hypothesized (v) that these scales would correlate well with ratings of dynamic risk of violence, harm or suicide, but less well with ratings of static, historical risk factors. (vi) We expected these items to correlate with other measures of met and unmet need. As a proxy for the intended function of these items and scales, we hypothesized (vii) that they would differ according to the level of leave the patient was currently allowed and (viii) according to whether the patient was currently in acute/high secure, medium secure, low secure/pre-discharge or community placements.

## Methods

### Study design

This is a cross-sectional ecological or naturalistic study employing routine outcome and clinical audit data on all patients in the National Forensic Mental Health Service for Ireland between the months of February and March 2010.

### Setting

At the time of the study the National Forensic Mental Health Service for Ireland had 93 secure in-patient beds, including eight for women, at the Central Mental Hospital. The male beds are in wards (units) organised into a coherent pathway through care. There is a 12 bed high secure male admission unit and a six bed high secure intensive care unit. Patients who are not discharged early back to prison or to their local psychiatric services progress to a 16 bed medium secure ward where pharmacotherapy aims to achieve remission from symptoms while the emphasis is on patient education regarding physical and mental health and substance misuse programmes. Patients progress from there to a second 16 bed medium secure unit where treatment programmes continue, emphasising meta-cognitive training, enhanced thinking skills, dialectic behaviour therapy or selected modules from that programme, anger management and education regarding healthy relationships. These programmes are designed in three phases, first to provide preliminary short educational modules, second, full manualised programmes in which patients are encouraged to engage and participate by bringing personal examples for group work. There is then a third, maintenance or refresher phase. Patients progress to a 15 bed pre-discharge unit which has open access to the grounds of the hospital (within a secure perimeter). The emphasis here is on the completion of programmes and occupational therapy aimed at optimising activities of daily living and preparation for return to the community. There is also a ten bed 'slow stream' low secure unit. The next step is to a ten bed 'hostel ward' in the grounds of the hospital within the secure perimeter. Patients there are mostly engaged in occupational and educational activities outside the hospital during the day, in preparation for discharge. There is also a six bed community residence which is staffed 24 hours a day. A the time of this survey, all patients in the community residence were legally categorised as on temporary leave from the hospital, returning to the hospital at intervals of between once a week and once a month. Women patients are currently accommodated in an 8 bed unit which is best described as high secure because of the staff to patient ratio and physical design. However programmes are individualised according to the stage of treatment and rehabilitation.

### Assessments

The study was approved by the local research, audit, ethics and effectiveness committee as part of the clinical governance audit programme for quality management at the hospital. All patients were assessed as part of routine outcomes assessments and clinical audit. All patients consented to those assessments that required interviews. Where assessments required the rating clinician to obtain information from a key informant this was either the primary nurse or key worker who had the longest current knowledge of the patient, or the consultant psychiatrist responsible for the care and treatment of the patient, as appropriate to the particular rating instrument.

### DUNDRUM-3 Programme Completion and DUNDRUM-4 Recovery instruments

The two structured professional judgment instruments the DUNDRUM-3 and DUNDRUM-4 are the products of an iterative drafting process, which has been described elsewhere [1]. The structured professional judgment instruments described here - the DUNDRUM-3 programme completion and DUNDRUM-4 recovery instruments are part of the 22nd revision of this draft. They form part of a suite of structured professional judgment instruments [2] along with the DUNDRUM-1 triage security instrument and DUNDRUM-2 triage urgency instrument, for assessing the need for admission and prioritisation of waiting lists. We have trained mental health professionals from psychiatry, nursing, psychology, social work and occupational therapy to use the DUNDRUM-3 and DUNDRUM-4 by guiding the use of the handbook followed by joint ratings of three prepared vignettes. This training takes two and a half hours and forms part of the induction for all new clinical staff, with optional top up training every six months.

### Rating Scales

The DUNDRUM-3 Programme Completion instrument [2] was completed by interviewing the key worker and by reference to the multi-disciplinary case notes. The DUNDRUM-4 [2] was completed in the same way. No patient interview was required. Ratings were made in February and March 2010 jointly by two (SO'D & MD) post-membership trainee psychiatrists (equivalent to U.S. fellows) who were trained in the use of the manual by HGK. They were blind to the ratings of other instruments made by colleagues. They also completed ratings for the DUNDRUM-1 triage security instrument [1,2]. Both can be rated together in an hour, less when the clinician already knows the patient.

The Positive and Negative Symptom Scale (PANSS) [13] and Global Assessment of Function (GAF) [14] were completed in February and March 2010 by different post-membership trainee psychiatrists who were blind to the ratings of DUNDRUM-3 and DUNDRUM-4. Face to face interviews were carried out and in addition primary nurses and treating psychiatrists were also consulted.

The HCR-20 rates historical (fixed), clinical and 'risk' (dynamic) items concerning the risk of violence to others [9] and the S-RAMM rates background (fixed), current and future (dynamic) risk factors for suicide and self harm [11]. These were rated by the treating consultant-led multi-disciplinary teams. All members of the multi-disciplinary teams were trained in the use of the HCR-20 and S-RAMM by accredited trainers. These were collated by ZA and ratings were checked for consistency by consulting with the clinicians who made the ratings, in February and March 2010.

The Camberwell Assessment of Need, Forensic Version (CANFOR) [15] was completed by the social worker members of each multi-disciplinary team who were trained in the use of the manual using a training video prepared in house. Ratings were coordinated for consistency by KMcD, a Masters-level psychiatric social worker in February and March 2010.

### Outcome Measures

The most valid outcome measure would be the subsequent movement of patients from higher to lower levels of therapeutic security or to the community, whether conditionally discharged or absolutely discharged. The data presented here are the basis for such a prospective study. To establish preliminary validity in this study, proxy outcome measures have been employed. All patients have been grouped according to whether at the time of the assessments they had been allowed no leave outside the hospital, accompanied leave or unaccompanied leave outside the hospital. Male patients were also grouped according to the ward or unit in which they were placed, since the units for men are arranged along a system of stratified therapeutic security, with the men's intensive care unit and admission unit combining as the first cluster of high secure environments; the second 'medium' cluster comprises two men's medium secure units each with progressively lower staff to patient ratios and less restrictive procedural security. This cluster also includes a longer term/slow stream low secure unit. The third cluster of pre-discharge/community units is the next step on the recovery pathway - from the medium term medium secure units patients progress to a medium term low secure unit then to a hostel ward within the secure perimeter of the hospital and then on to a high support 24 hour nurse staffed residence in the community, these last two constituting a pre-discharge/community cluster. Those who made decisions about leave and placement within the hospital made those decisions prior to the rating of patients using these scales and the decision makers were therefore blind to the ratings.

### Statistics

All data were entered in SPSS-16 [16] and stored in anonymised form. Correlations were calculated using the Spearman rank correlation coefficient, a non-parametric measure to avoid the assumption of normal distribution. Scale properties were assessed using factor analysis and Cronbach's alpha statistic for internal consistency. Inter-rater reliability was assessed using Spearman's rank correlation for scale scores. For individual items, the kappa statistic was used where it could be calculated and for all items, the Spearman rank correlation coefficient and the X^2 ^linear by linear coefficient. Groups were compared using univariate analysis of variance. Items and outcomes were compared using univariate analysis of variance and Chi-squared test.

## Results

### Patients

There were 95 patients in the hospital when data were gathered in February and March 2010, 8 women and 87 men. The mean age was 40.9 years (95% confidence interval 38.4 to 43.5). The mean length of stay was 7.2 years (95% confidence interval 5.3 to 9.2 years). The acute/high secure cluster included the women's unit, the men's admission unit and an intensively staffed men's unit, 25 (26%) patients in all. In some of the analyses that follow, only male patients are included because women are part of a separate recovery pathway. The medium cluster units comprised of two medium secure units for men and one slow-stream or longer term low secure unit, 40 (42%) patients in all. The pre-discharge and community service had 30 (32%) patients.

Primary diagnosis was schizophrenia in 68 (72%), schizoaffective disorder in 8 (8%), bi-polar affective disorder in 11 (12%), psychotic depression in 5 (5%) and intellectual disability in 3 (3%). There were 25 (26%) patients transferred from prisons, 8 (8%) detained because unfit to stand trial, 46 (48%) not guilty by reason of insanity and 16 (17%) who were detained under the civil mental health act and transferred from local psychiatric services.

No leave outside the hospital was permitted for 55 (58%) patients, 20 (21%) were allowed leave accompanied by staff and 20 (21%) were allowed unaccompanied leave as part of their pre-discharge rehabilitation programmes.

### Inter-Rater Reliability

The ratings of 21 of the patients using the DUNDRUM-3 and DUNDRUM-4 by the researchers (SO'D and MD) were replicated over the period of data collection by HGK, who was blind to the ratings of SO'D and MD. The kappa statistic could be calculated for 7 of the 13 items and ranged between 0.44 and 0.77 (all p < 0.001). For all 13 items the X^2 ^linear by linear association was greater than 6.9 (df = 1, p < 0.01) and the Spearman rank correlation coefficient was greater than 0.51 (range 0.51 to 0.95, all p < 0.02). For items rated 0 to 4, random agreement between two raters can be expected in 5 out of 25 'cells' i.e. 20% of cases. a probability of 0.2. For the DUNDRUM-3 programme completion items, there was exact agreement between the two raters for item 1 in 19 of 21 cases (90%, binomial exact probability p < 0.001), exact agreement for items 2 and 3 in 16/21 (76%, p < 0.001), for item 4 in 13/21 (62%, p < 0.001), for items 5 and 6 in 18/21 (86%, p < 0.001) and exact agreement for item 7 in 17/21 (81%, p < 0.001). For the DUNDRUM-4 recovery items there was exact agreement between the two raters for item 1 in 14/21 cases (67%, p < 0.001), for item 2 in 16/21 (76%, p < 0.001), for item 3 in 17/21 (81%, p < 0.001), for item 4 in 13/21 (62%, p < 0.001), for item 5 in 18/21 (86%, p < 0.001) and for item 6 in 15/21 (71%, p < 0.001).

### Internal consistency

The seven programme completion items were subjected to a principle components factor analysis. All statistics are for 95 patients. Initial extraction yielded one factor with an Eigen value of 4.65 accounting for 66% of the variance and other factors had Eigen values less than 1. All seven items loaded positively on the first factor (r values all >0.724). Cronbach's alpha statistic for the seven Programme Completion items was 0.911. Cronbach's alpha if any one item was deleted was in the range 0.886 to 0.911.

The six recovery items were subjected to a principle components factor analysis. Initial extraction yielded one factor with an Eigen value of 3.9 accounting for 66% of the variance and other factors had Eigen values less than 1. All six items loaded positively on this first factor (r values all >0.75). Cronbach's alpha statistic for the six recovery items was 0.890. Cronbach's Alpha if any one item was omitted was in the range 0.845 to 0.885 except for item 6 'victim sensitivities' deletion of which led to an increase of the alpha statistic to 0.982, a negligible change.

Factor analysis on all thirteen items together yielded two components with Eigen values greater than 1. The first had an Eigen value of 7.7 and accounted for 59% of the variance while the second had an Eigen value of 1.3 and accounted for 9.8% of the variance. All thirteen items loaded strongly positively on the first component (all r > 0.575), while all the recovery items loaded positively on the second component with five of the seven programme completion items loading negatively on the second component. Only two programme completion items, programme completion item 4 'problem behaviours' and programme completion item 7 'family and social networks' loaded positively on the second component. Cronbach's Alpha for the combined scale of thirteen items was 0.94 and only one item if omitted lead to an increase in the Alpha statistic. This was recovery item 6 'victim sensitivity', omission of which gave an alpha of 0.941, a negligible increase. Because of the face validity of the two scales as distinct in content and because of the result of the factor analysis for all thirteen items, it appears reasonable to treat them as separate for validation purposes, while also accepting that they could be used as a single score for validation purposes. The DUNDRUM-3 Programme Completion and DUNDRUM-4 Recovery scores correlated with each other (Spearman r = +0.730, p < 0.001)

### Possible confounders

Age correlated poorly with the DUNDRUM-3 Programme completion score (Spearman r = -0.205, p = 0.047) and did not correlate significantly with the DUNDRUM-4 Recovery score (r = -0.205, NS) or the total score for all 13 items (r = -0.175, NS).

Length of stay correlated inversely but weakly with the DUNDRUM-3 Programme completion score (r = -0.337, p < 0.001), with the DUNDRUM-4 Recovery score (r = -0.352, p < 0.001) and with the total for all 13 items (r = -0.348, p < 0.001).

Men and women did not differ significantly for mean scores in DUNDRUM-3 Programme completion, DUNDRUM-4 Recovery or combined scores.

The PANSS positive symptom score correlated 0.516 with the DUNDRUM-3 programme completion score and 0.656 with the DUNDRUM-4 Recovery score. The PANSS negative symptom score correlated 0.525 with the DUNDRUM-3 programme completion score and 0.487 with the DUNDRUM-4 recovery score. The PANSS general symptom score correlated 0.506 with the DUNDRUM-3 programme completion score and 0.495 with the DUNDRUM-4 recovery score. The PANSS total score correlated 0.574 with the DUNDRUM-3 programme completion score and 0.596 with the DUNDRUM-4 recovery score. The Global Assessment of Function (GAF) score correlated inversely -0.650 with the DUNDRUM-3 programme completion score and -0.673 with the DUNDRUM-4 recovery score. All correlations with PANSS scales and GAF were statistically significant p < 0.001, n = 95.

### Cross-validation with measures of risk and need for therapeutic security

The DUNDRUM-3 programme completion score correlated with the DUNDRUM-1 triage security score, a measure of need for therapeutic security (r = 0.346, p < 0.001) as did the DUNDRUM-4 recovery score (Spearman r = 0.444, p < 0.001).

The DUNDRUM-3 Programme Completion score correlated with the HCR-H score (the sum of the 10 'historical' or fixed risk factors) r = 0.480; with the HCR-C score (the sum of the five 'clinical' or current risk factors) r = 0.637; with the HCR-R score (the sum of the five 'risk' or future risk factors) r = 0.519; with the HCR-dynamic score (the sum of the 'C' and 'R' risk factors) r = 0.629 and with the HCR-20 total score r = 0.686 (all significant p < 0.001, n = 95).

The DUNDRUM-4 Recovery score correlated with the HCR-20 H sub-scale r = 0.446 and with the HCR-C score r = 0.731; with the HCR-R r = 0.533; with the HCR-dynamic items (C and R combined) r = 0.704; and with the HCR-20 total score r = 0.713 (all significant p < 0.001, n = 95). Because the fifth item of the DUNDRUM-4 recovery score is itself strongly dependent on the dynamic items of the HCR-20, a score was calculated for the other five of the DUNDRUM-4 recovery items only. This correlated with the HCR-H score r = 0.447; with the HCR-C score r = 0.725; with the HCR-R score r = 0.519; with the HCR-dynamic score r = 0.694; and with the HCR-20 total score r = 0.705 (all significant p < 0.001, n = 95), the omission making little difference.

The DUNDRUM-3 Programme Completion score correlated with the S-RAMM Background (historical, fixed) score r = 0.263 (p = 0.05); with the S-RAMM Current score r = 0.529; with the S-RAMM Future score 0.451; with the S-RAMM dynamic score (the sum of the S-RAMM current and future scores) r = 0.553; and with the S-RAMM total score r = 0.556 (all significant p < 0.001 except where indicated, n = 95).

The DUNDRUM-4 Recovery score correlated with the S-RAMM background score r = 0.197 (not significant); with the S-RAMM Current score r = 0.613; with the S-RAMM Future score r = 0.609; with the S-RAMM dynamic score r = 0.702; and with the S-RAMM total score r = 0.628 (all significant p < 0.001, n = 95 except where indicated).

### Cross-Correlation With the CANFOR, a Measure of Met, Unmet and Total Need

Using the CANFOR, The DUNDRUM-3 programme completion score did not correlate significantly with the patient self-rated met needs (Spearman r = -0.11, NS), it did correlate with the patient self-rated unmet needs (r = 0.33, p = 0.002) and did not correlate with the patient rated total needs (r = 0.114, NS). For staff ratings, met needs did not correlate significantly with the DUNDRUM-3 programme completion score (r = 0.09, NS) but the DUNDRUM-3 did correlate with unmet needs r = 0.50, (p < 0.001) and total needs r = 0.36 (p < 0.001).

Comparing the CANFOR and the DUNDRUM-4 recovery score, patient self-ratings for met need did not correlate (r = -0.182, NS), patient rated unmet needs correlated modestly (r = 0.253, p = 0.019) while patient rated total needs did not correlate (r = 0.019, NS). Staff ratings of met need did not correlate with the DUNDRUM-4 score (r = -0.022, NS), though staff-rated unmet need correlated 0.501 (p < 0.001) while staff-rated total need correlated weakly (r = 0.238, p = 0.02).

### Leave

Patients were divided into those who had no leave outside the hospital, those who had leave outside the hospital only when accompanied by staff and those who had unaccompanied leave outside the hospital. Because the fourth item in the DUNDRUM-4 recovery scale is largely determined by the level of leave, this was recalculated for the five other items only - referred to here as the DUNDRUM-4 RL score.

Table 1 shows that the DUNDRUM-3 programme completion score was significantly lower for those who were allowed unaccompanied leave (ANOVA F = 38.1, df = 2, p < 0.001). Bonferroni post-hoc tests for multiple comparisons showed that those with unaccompanied leave had significantly lower scores than those with no leave or accompanied leave. This was also true for the DUNDRUM-4 recovery score (ANOVA F = 76.8, p < 0.001), post hoc tests showed that those with unaccompanied leave and accompanied leave both had significantly lower scores that those who had no leave p < 0.001 and p = 0.014, and held also when the leave item was excluded - DUNDRUM-4 RL, (ANOVA F = 56.6, df = 2, p < 0.001) post hoc tests demonstrated that those with unaccompanied leave had lower scores than those with accompanied leave (p < 0.001) or no leave (p < 0.001) while those with accompanied leave also had lower scores than those with no leave (p = 0.009).

**Table 1:** DUNDRUM-3 and DUNDRUM-4 according to level of leave outside hospital.

		No leave	Accompanied leave	Unaccompanied leave	All	ANOVA df = 2
	N	55	20	20	95	F/p

DUNDRUM-3,	mean	18.9	17.2	7.1	16.1	38.1/ 0.001
		
programme completion	95% CI	17.6 - 20.3	14.5 - 19.9	4.7 - 9.4	14.6 - 17.5	

DUNDRUM-4, Recovery	mean	19.1	16.4	7.3	16.1	76.8/ 0.001
		
	95% CI	18.2 - 20.1	14.4 - 18.3	5.4 - 9.1	14.8 - 17.3	

DUNDRUM-4,	mean	15.6	14.2	6.2	13.3	56.6/ 0.001
		
RL4-(omits Leave)	95% CI	14.7 - 16.5	12.4 - 15.9	4.4 - 7.9	12,3 - 14.3	

Mean and 95% confidence intervals. Note DUNDRUM-4, RL4 is the total of only five items, omitting leave.

### Stratification along the recovery pathway - clusters

Because the women's unit is not part of the same recovery pathway as the arrangement of wards and units providing for men, the arrangement of wards into three clusters (acute & high secure, medium secure and pre-discharge) refers only to the 87 male patients. Table 2 shows that the DUNDRUM-3 programme completion score was significantly different when each of the three stages were compared with each other (ANOVA F = 45.9, df = 2, p < 0.001, Bonferroni post hoc tests pre-discharge significantly less than acute/high secure p < 0.001 and medium secure p < 0.001, medium secure significantly less than acute/high secure p < 0.05). The DUNDRUM-4 recovery score was significantly lower only for those in the pre-discharge cluster (ANOVA F = 66.8, df = 2, p < 0.001, post-hoc test pre-discharge less than medium < 0.001 and acute/high secure p < 0.001).

**Table 2:** DUNDRUM-3 and DUNDRUM-4 according to place in the recovery pathway

		Acute/high secure cluster	Medium Secure Cluster	Pre-discharge/community cluster	ANOVA
	N	17	40	30	F/p df = 2

DUNDRUM-3,	mean	22.2	18.7	9.4	45.9/ 0.001
		
programme completion	95% CI	20.2-24.1	17.3 - 20.1	7.2 - 11.7	

DUNDRUM-4, Recovery	mean	19.4	19.7	9.9	61.2/ 0.001
		
	95% CI	17.8 - 20.9	18.8 - 20.5	7.9 - 11.9	

Mean and 95% confidence intervals

### Stratification along the recovery pathway - by units

Table 3 shows that static measures such as the DUNDRUM-1 triage security score, the HCR-20 'H' score (sum of historical items) and S-RAMM 'B' score (sum of background items) differ between units to a limited extent, with no clear pattern other than the accumulation of those with the highest fixed historical risk profile or risk factors in the intensive care unit while the pre-discharge units accumulate those with lower scores. The dynamic risk scores for the HCR-20 and S-RAMM present a clearer pattern of stratification from intensive care and admission units to pre-discharge. The strongest and most consistent stratification was found for the DUNDRUM-3 programme completion (ANOVA F = 45.9, df = 6, p < 0.001) and DUNDRUM-4 recovery scores (ANOVA F = 33.9, df = 6, p < 0.001) with falling scores from high secure/intensive care through medium secure units to low secure, pre-discharge and community high support.

**Table 3:** Cross-sectional stratification of patients along the recovery pathway - DUNDRUM-1 triage security, DUNDRUM-3 programme completion, DUNDRUM-4 recovery, HCR-20 historical (fixed), HCR-20 dynamic ('clinical' and 'risk' items), S-RAMM background (fixed) and S-RAMM dynamic ('current' and 'future' items).

		high secure-intensive care	Male admission/high secure	Male medium secure-1	Male medium secure-2	Long term low security	Male low secure rehab	Male pre-discharge/community high support	ANOVA
N		7	10	14	16	10	15	15	F/df = 6/p

DUNDRUM-1 triage security score	Mean	33.4	26.8	30.9	31.0	31.0	29.0	26.8	4.1/0.02
		
	95% CI	29.6-37.3	22.9-30.7	28.3-33.5	28.6-33.4	29.0-33.0	26.2-31.8	22.9-30.7	

DUNDRUM-3 programme completion	Mean	23.3	21.4	20.4	17.3	18.6	13.0	5.8	45.9/0.001
		
	95% CI	19.5-27.1	18.9-23.9	17.9-22.9	15.4-19.1	14.6-22.6	10.3-15.7	3.1-8.5	

DUNDRUM-4 Recovery	Mean	20.7	18.4	19.9	19.4	19.7	13.4	6.5	33.9/0.001
		
	95% CI	18.5-22.9	16.0-20.8	18.2-21.5	18.2-20.7	17.7-21.7	10.6-16.2	4.9-8.0	

HCR-H	Mean	15.3	13.1	14.4	13.5	13.6	11.8	10.2	6.5/0.002
		
	95% CI	13.9-16.7	10.3-15.9	10.9-17.8	11.9-17.8	11.8-15.5	10.4-13.2	8.7-11.7	

HCR-dynamic	Mean	14.1	8.9	8.7	6.8	7.5	3.5	2.5	26.0/0.001
		
	95% CI	10.4-17.9	5.5-12.3	6.2-11.2	4.5-8.9	4.9-10.1	2.0-4.9	1.2-3.8	

S-RAMM-B	Mean	11.9	9.0	9.8	10.9	9.5	9.7	8.1	2.0/0.14(NS)
		
	95% CI	9.3-14.4	7.2-10.8	8.7-10.9	9.6-12.2	7.5-11.5	7.8-11.7	6.6-9.6	

S-RAMM-dynamic	Mean	13.8	11.5	11.6	11.9	11.5	8.1	4.0	24.0/0.001
		
	95% CI	10.9-16.8	9.2-13.8	8.9-14.2	9.8-13.9	8.2-14.8	10.9-16.8	2.9-5.1	

Bonferroni post hoc tests show that for the DUNDRUM-1, only the intensive care and the pre-discharge/community groups differed significantly (p = 0.021). For the DUNDRUM-3 and DUNDRUM-4, the low secure rehab and pre-discharge/community groups differed significantly from all other groups and from each other. For HCR-H, the medium secure unit 1 differed from the pre-discharge and community group p < 0.05. For HCR-dynamic the intensive care unit had a significantly higher mean score than all other units except the long term low secure unit, the low secure rehab unit was significantly lower than the intensive care unit, male medium secure unit 2 and the long term low secure unit, while the pre-discharge/community group was lower than all other units except the low secure rehab unit. S-RAMM dynamic scores were significantly lower in the pre-discharge/community group than for any other unit and the low secure rehab unit was significantly lower than the intensive care unit.

Table 4 shows that the Global Assessment of Function (GAF) increases progressively from the intensive care and admission units through medium secure and low secure to pre-discharge and community units (ANOVA F = 12.0, p < 0.001). PANSS positive, PANSS negative, PANSS general and PANSS total scores also stratified, though less consistently. Table 4 also shows that for the CANFOR, staff ratings of unmet need differed significantly across the recovery pathway as expected, (F = 5.9, p < 0.001) but neither staff-rated met needs nor staff-rated total needs differed significantly. Patient self-rated needs, met, unmet and total did not differ significantly.

**Table 4:** Cross-sectional stratification of patients along the recovery pathway - global function, symtoms and Camberwell Assessment of Need, forensic version (CANFOR).

		high secure-intensive care	Male admission/high secure	Male medium secure-1	Male medium secure-2	Long term low security	Male low secure rehab	Male pre-discharge/community h1gh support	ANOVA Df = 6
N		7	10	14	16	10	15	15	F/p

GAF	mean	34.6	45.8	47.4	54.4	47.8	60.5	73	13.8/0.001
		
	95% CI	20.3-48.8	35.3-56.3	39.8-55.1	49.2-59.5	40.3-55.3	56.7-64.4	67.6-78.4	

PANSS positive	mean	21.4	13.6	17.9	13.3	14.6	11.3	8.9	5.4/0.001
		
	95% CI	13.4-29.5	11.1-16.1	13.7-22.1	10.5-16.2	8.7-20.5	8.6-13.9	7.1-10.6	

PANSS negative	mean	23.6	22.3	21.9	15.6	21.1	17.0	10.9	5.9/0.001
		
	95% CI	16.3-30.8	16.1-28.5	17.4-26.4	12.6-18.5	16.1-26.2	13.4-20.6	8.7-12.9	

PANSS general	mean	37.6	31.5	32.4	28.8	29.1	25.9	21.9	3.5/0.008
		
	95% CI	27.0-48.1	24.7-38.3	27.4-37.5	23.6-34.0	21.8-36.4	21.5-30.4	18.8-36.4	

PANSS total	mean	82.6	67.4	72.3	57.7	64.8	54.2	41.6	5.6/0.001
		
	95% CI	57.9-107.2	54.8-79.9	60.2-84.4	47.9-67.5	48.4-81.2	44.1-64.3	35.7-47.5	

CANFOR staff met needs	mean	7.0	6.1	6.1	8.1	6.9	6.5	7.0	0.9/0.5 (NS)
		
	95% CI	4.2-9.8	4.6-7.6	4.1-7.9	6.6-9.5	4.8-8.9	5.4-7.5	5.7-8.3	

CANFOR staff unmet needs	mean	5.7	3.1	4.4	2.9	2.7	1.4	1.0	5.9/0.001
		
	95% CI	3.5-7.9	1.5-4.7	2.2-6.7	2.0-3.9	1.2-4.2	0.8-2.0	0.4-1.6	

CANFOR staff total needs	mean	12.7	9.2	10.5	11.0	9.6	7.9	8.0	2.2/0.052 (NS)
		
	95% CI	8.7-16.7	6.3-12.1	7.3-13.7	9.1-12.9	6.2-12.9	6.8-8.9	6.6-9.4	

Bonferroni post hoc tests show that for the GAF, the pre-discharge/community group had significantly higher scores than all other units except the male low secure rehab unit while the intensive care unit had significantly lower scores than all but the admission high secure and medium secure unit 1. The PANSS positive score was significantly higher when the intensive care unit was compared with the low secure rehab and pre-discharge/community groups while the pre-discharge/community group had lower scores than the intensive care unit and the medium secure unit 1. PANSS negative scores were lower for the pre-discharge/community group than for the intensive care unit, admission/high secure unit, medium secure unit 1 and long term low secure unit. For PANSS general score, the pre-discharge/community group had a lower mean score than the intensive care unit or the medium secure unit 1. PANSS total score was higher for the intensive care unit than for the male low secure rehab and pre-discharge/community units while the pre-discharge and community group had a lower mean PANSS total score than the intensive care unit and the medium secure unit 1. CANFOR staff unmet needs were greater for medium secure unit 1 than the low secure rehab unit, the intensive care unit had a higher score than the low secure rehab unit and the pre-discharge/community group and the pre-discharge/community group had a lower mean score than the intensive care unit and medium secure unit 1.

### DUNDRUM-3 and DUNDRUM-4 Individual Items and Proxy Outcomes

Tables 5 and 6 show that each item of the DUNDRUM-3 programme completion instrument and DUNDRUM-4 recovery instrument differed significantly across the recovery pathway clusters and according to the level of leave allowed at the time the ratings were made. Note that for level of leave, the DUNDRUM-4 recovery item 4 'leave' has an extremely high statistical significance because the definition makes this almost a circular measure.

**Table 5:** Item to outcome: DUNDRUM-3 and DUNDRUM-4 according to level of leave

	No leave	Accompanied leave	Unaccompanied leave	ANOVA Df = 2	Chi-Squared Df = 8
N	55	20	20	F	X^2^

PC-1 Physical health	2.7(0.9)	2.6(1.2)	1.4(1.1)	12.8	31.0

PC-2 Mental health	3.0(0.9)	2.9(0.8)	1.4(1.2)	19.5	33.9

PC-3 Drugs and Alcohol	2.5(1.3)	2.5(1.4)	0.9(0.9)	13.2	24.3

PC-4 Problem behaviours	3.4(0.9)	2.8(1.1)	1.1(1.1)	39.9	52.9

PC-5 Self-care and activities of daily living	2.6(0.9)	2.1(0.9)	0.9(0.9)	22.7	42.8

PC-6 Education, Occupation and Creativity	2.4(1.0)	1.9(0.9)	0.8(0.9)	22.0	41.9

PC-7 Family and Social Networks	2.4(1.0)	2.7(1.1)	0.8(1.0)	21.1	45.4

R-1 Stability	2.9(1.2)	2.4(1.1)	1.3(0.8)	17.8	35.6

R-2 Insight	2.9(0.9)	2.7(1.0)	1.2(0.9)	23.7	51.2

R-3 Rapport and Working Alliance	3.1(0.8)	2.7(0.9)	1.4(0.8)	31.6	54.3

R-4 Leave	3.6(0.7)	2.1(0.8)	1.1(0.4)	116.7	92.8

R-5 HCR-20 Dynamic Items	3.8(0.6)	3.5(1.0)	1.5(1.1)	60.5	54.8

R-6 Victim issues	2.8(1.1)	2.9(0.9)	0.9(1.3)	23.4	43.7

Means and standard deviations. All significant p < 0.001.

**Table 6:** Item to Outcome: DUNDRUM-3 and DUNDRUM-4 according to place in the recovery pathway

	Acute/high secure cluster	Medium Secure Cluster	Pre-discharge/community cluster	ANOVA Df = 2	Chi-Squared Df = 8
N	17	40	30	F	X^2^

PC-1 Physical health	3.1(0.6)	2.8(0.8)	1.6(1.1)	19.4	32.2

PC-2 Mental health	3.4(0.6)	2.9(0.9)	1.8(1.2)	22.4	33.5

PC-3 Drugs and Alcohol	3.4(0.6)	2.5(1.3)	1.3(1.1)	21.3	35.6

PC-4 Problem behaviours	3.7(0.6)	3.3(0.8)	1.6(1.4)	33.2	47.7

PC-5 Self-care and activities of daily living	2.9(0.9)	2.6(0.8)	1.1(0.8)	37.6	45.5

PC-6 Education, Occupation and Creativity	3.1(0.9)	2.3(0.9)	1.0(0.9)	28.4	50.2

PC-7 Family and Social Networks	2.7(1.1)	2.3(0.9)	1.2(1.4)	12.0	40.5

R-1 Stability	3.6(1.0)	2.9(1.0)	1.4(0.9)	22.9	53.7

R-2 Insight	2.8(0.9)	3.2(0.9)	1.6(1.0)	25.9	48.8

R-3 Rapport and Working Alliance	3.0(0.7)	3.2(0.7)	1.6(1.1)	33.6	55.5

R-4 Leave	3.4(0.6)	3.4(0.9)	1.7(1.1)	32.4	60.4

R-5 HCR-20 Dynamic Items	3.8(0.4)	3.9(0.3)	2.0(1.4)	47.2	50.0

R-6 Victim issues	2.8(1.3)	3.0(0.9)	1.6(1.5)	11.5	29.3

Means and standard deviations. All significant p < 0.001.

## Discussion

We have constructed two structured professional judgement instruments specifically to cover those factors identified as likely to be relevant to the decision to allow increasing amounts of leave from the hospital and to move from one level of therapeutic security to a lower level, or to the community. The two instruments are conceptually distinct. The DUNDRUM-3 allows clinicians to make ratings of the extent to which a patient has successfully completed treatment programmes in a range of domains relevant to forensic mental health needs. The DUNDRUM-4 allows clinicians to rate aspects of the patient's recovery relevant to successful risk management. A factor analysis lends some support to a distinction between the two, though in practice the two scales appear to be largely measuring the same statistical tendency. The two scales have acceptable inter-rater reliability and internal consistency. We have shown that the two instruments, the DUNDRUM-3 programme completion items and the DUNDRUM-4 recovery items correlate modestly with the criteria for admission to therapeutically secure hospitals (the DUNDRUM-1) and are distinct from those criteria. We have shown in this cross-sectional study that the DUNDRUM-3 and DUNDRUM-4 appear to vary between patient groups in the same way as dynamic measures of risk, symptoms of mental illness and global function. All of this supports the validity of the use of these two structured professional judgement instruments when making decisions regarding the progression of forensic mental health patients from high or medium security to low security and on to conditional or absolute discharge in the community.

We believe that both the programme completion items and recovery items explicitly measure domains related to personal recovery, with a rating system that explicitly incorporates the trans-theoretical model [17,18], a five stage model of recovery [19,20] and Maslow's hierarchy of needs [21]. We have added a system for rating engagement [2]. Both the programme completion items and the recovery items take account of social and community factors (e.g. item PC7 social and family networks, item R6 victim issues), which go beyond individual risk or psychopathological factors and also go beyond clinician and consumer perspectives. We believe this reflects the reality of how decision makers make these decisions - not just the treating clinicians, but also mental health tribunals and where relevant Justice ministries. We believe these are legitimate considerations and decisions made without considering them would be flawed, leading to failed returns to the community which would have adverse consequences for the patient as well as other stake holders including victims.

The scoring framework for each item is constructed so that '4' indicates that the person is not ready for a move to a less secure place, '3' indicates readiness for a move from high to medium therapeutic security, '2' from medium to low secure, '1' from low security to an open or community placement and '0' indicates readiness for an absolute discharge. The mean scores, if divided by the number of items, yield an estimate of 'average' readiness to move. This gives an indication of the appropriateness of placements within a service overall. Table 2 can be read in this way to show that the average score for men in the acute/high secure cluster on the DUNDRUM-3 was 3.2 and on the DUNDRUM-4 was also 3.2. The medium cluster had an average score of 2.7 for the DUNDRUM-3 and 3.3 for the DUNDRUM-4 while the pre-discharge and community cluster had an average score of 1.3 for the DUNDRUM-3 and 1.7 for the DUNDRUM-4. Table 3 allows a more precise calculation for the 15 patients in the pre-discharge hospital hostel ward and the community high support residence, who had an average score of 0.8 on the DUNDRUM-3 and 1.1 on the DUNDRUM-4. These averages indicate the extent to which a ward or unit is being used as intended. Another way to read these average scores for a ward or a cluster of wards such as the acute/high secure cluster would be to consider how many individuals in the acute/high secure cluster scored above an average of '3' - 11/16 (69%) using the DUNDRUM-3 programme completion items, 15/16 (94%) using the DUNDRUM-4 recovery items. A further analysis might ask how many had scored '4' on any one item (all 16 male patients in the acute/high secure cluster).

When making a decision about an individual, a single high-scoring item may outweigh an individual's low 'average' score. Such an assessment should then be used clinically to prioritise that issue in the treatment plan, and to engage the patient in motivational work on that issue.

## Conclusions

We have shown that these two structured professional judgement instruments meet many of the criteria for the validity of risk assessment instruments [22]. These are not, however, intended for use as risk assessment instruments. The decisions they help to structure are correlated to some extent with measures of dynamic risk of harm to others and to self, but the content of items is for the most part very different because the purpose of the ratings is to make decisions about moves between levels of therapeutic security. Our current clinical practice is to assess the HCR-20 and S-RAMM, PANSS and GAF, DUNDRUM-3 and DUNDRUM-4 every six months prior to case conferences and when recommending leave or moves to less secure settings We believe these two structured professional judgement instruments fulfil many of the criteria set out for personal recovery measures [23,24] because they explicitly measure domains related to personal recovery, are brief and easy to use, take a consumer perspective while also taking the clinician and societal perspectives, yield quantitative data, have been scientifically scrutinised, demonstrate sound psychometric properties and are acceptable to consumers. It follows however that the true criterion measure of validity for these two structured professional judgement instruments is the subsequent movement of patients from higher to lower levels of therapeutic security or to the community. This data represents the first stage of a prospective study in which movements between levels of therapeutic security and discharge will be the outcomes.

We would welcome the piloting and validating of these instruments in other jurisdictions. We believe the handbook is sufficient to use the instruments reliably but we are willing to assist any clinicians or academics wishing to use these instruments for practice, audit or research.

## Competing interests

The authors declare that they have no competing interests.

## Authors' contributions

SO'D and MD rated patients using the DUNDRUM-3 and DUNDRUM-4 and collated the database with ZA; ZA rated patients using the PANSS and GAF. ED and KMcD coordinated and collated ratings of HCR-20 and S-RAMM. KMcD collated the CANFOR ratings and contributed to the DUNDRUM-4. ED helped coordinate the rating of the DUNDRUM-3. HGK wrote the first draft of the handbook, designed the study and carried out the data analysis. All contributed to the authorship of the paper.

## Supplementary Material

Additional file 1 **Section One: DUNDRUM-3: Programme Completion Items (pp. 1 - 29)**. A structured seven item professional judgement instrument devised to consistently assess risk factors and security requirements at every stage of patient recovery and treatment. **Section Two: DUNDRUM-4: Recovery Items (pp. 30 - 43)**. A structured six item professional judgement instrument to assist the decision to move patients from higher to lower levels of therapeutic security. Click here for file
