# Supplementary material for: The DUNDRUM Quartet: validation of structured professional judgement instruments DUNDRUM-3 assessment of programme completion and DUNDRUM-4 assessment of recovery in forensic mental health services
Source: BMC Res Notes. 2011 Jul 3;4:229. doi: 10.1186/1756-0500-4-229 (PMC3146857; doi:10.1186/1756-0500-4-229)
Supplement: Additional file 1 — Section One: DUNDRUM-3: Programme Completion Items (pp. 1 - 29). A structured seven item professional judgement instrument devised to consistently assess risk factors and security requirements at every stage of patient recovery and treatment. Section Two: DUNDRUM-4: Recovery Items (pp. 30 - 43). A structured six item professional judgement instrument to assist the decision to move patients from higher to lower levels of therapeutic security. [file 1756-0500-4-229-S1.DOC]

# Additional files

Additional file 1 –.

**DUNDRUM-3: Programme Completion Items:**

For those discharged from a specialised forensic mental health service or moved from a higher to a lower level of therapeutic security, it is reasonable to expect that they would have completed programmes relevant to the risk items that required their original admission to the service. There should be a relationship between completion of the stages of these treatment programmes and progress from admission / high secure units to medium secure and on to rehabilitation and recovery (minimum security, pre-discharge) units and community follow-up. In devising this structured professional judgement instrument and the companion DUNDRUM-4 recovery items, we have been greatly influenced by the concept underpinning the HCR-20 Risk Management Manual (Douglas et al 2001). In practice we believe the items in the DUNDRUM-3 Programme Completion instrument will consistently address the risk factors identified in the course of risk assessment as well as in the assessed need for security.

Our starting point has been the proposition that remission of symptoms is not the same as recovery (Andreasen et al 2005) Recovery can be described in terms of stages and processes (Andresen et al 2003, Weeks et al 2010).

Five Pillars of Treatment

The programme completion items reflect the organisation of treatment programmes in practice, according to five ‘pillars’ of treatment: physical health, mental health, drugs and alcohol recovery, problem behaviours (offence related behaviour) and a fifth broad category that includes social, family and occupational life (Gill et al in press).

Taken together these five pillars are intended to cover the domains of health defined by the WHO (1946) “*Health is a state of complete physical, mental and social well-being and not merely the absence of disease or infirmity*”. A more recent definition from the WHO (1986), The Ottawa Charter for Health Promotion holds that health is *"a resource for everyday life, not the objective of living. Health is a positive concept emphasizing social and personal resources, as well as physical capacities."* (see also Jadad & O’Grady 2008).

There are existing research instruments and clinical rating scales that cover similar issues, often in the context if needs assessment research in forensic settings (Cohen & Eastman 2000). The TAPS project used the social behaviour schedule (Wykes et al 1986) and this has been used as part of needs assessment in forensic populations in different jurisdictions (Pierzchniak et al 1999, O’Neill et al 2003). We have shown (Pillay et al 2008) that the more recent research and clinical instruments for assessing treatment need such as the CANFOR (Thomas et al 2003) and HoNOS-SECURE (Sugarman & Walker 2004) appear to reflect differences in levels of met and unmet need for patients at different levels of therapeutic security – admission/high secure units, medium secure units and rehabilitation and pre-discharge units. Although these scales did differ significantly as patients progressed, the differences were small in absolute terms and confidence intervals overlapped.

There is good evidence that the HCR-20 clinical items demonstrate a similar pattern of stratification along the recovery pathway (Dernevik et al 2002, Muller-Isberner et al 2007) along with measures of mental state and global function (Pillay et al 2008) such as the PANSS (Kay et al 1987) and GAF (American Psychiatric Association 1994). Again we recommend that the DUNDRUM-3 & 4 items should be used with the HCR-20 or other risk assessment instruments. These scales measure something complimentary to risk and are not intended as risk assessments.

We have collated the content of existing scales such as the CANFOR and HoNOS and based on our experience of them, added items that we believe are relevant to the relationship between treatment, recovery and changing security need.

The rating scales for the recovery items include elements of Maslow’s (1943) hierarchy of needs and motivation. The programme completion stages referred to at level ‘4’ are mostly sufficient for physiological needs at best. Level ‘3’ should have elements of safety concerning the basics of life. Level ‘2’ concerns friendship and family relationships. Level ‘1’ aspires to self-esteem, confidence and social standing. Level ‘0’ emphasises the additional aspects of self-actualisation – morality, creativity, problem solving, acceptance of facts. While modern theorists tend towards the view that these needs are universal rather than hierarchical, the progression from need for basic care to autonomy fits well with the recovery model.

These rating items also include elements of the trans-theoretical model or stages of change (Prochaska & DiClemente 1983, DiClemente et al 1991) organised into five stages, starting with pre-contemplation (rated ‘4’), contemplation, preparation (rated ‘3’), action (rated ‘2’), maintenance (rated ‘1’ or ‘0’), with motivational work concerning ambivalence and decisional balance.

‘Engagement’ should be demonstrated through more than simply having attended all sessions of a programme. Engagement should include evidence that the person has benefitted from the programme. Evidence of engagement and benefit at its most basic would include passive participation, at the next level would include evidence of active engagement with retained information, changed attitudes and altered behaviour. Evidence of positive engagement includes showing the ability to personalise the content by giving examples of one’s own experiences relating to her/himself that are relevant to the content of the programme. Successful completion should mean having attended at least 90% of scheduled sessions in a programme during which the patient has actively participated. Those delivering programmes must therefore have time to complete reports on programme completion and there must be some system for outcome assessment.

Recovery can be described as five stages (Andresen et al 2003, Weeks et al 2010) – ‘moratorium’ a stage of hopelessness and self-protective withdrawal; awareness, the realisation that recovery and a fulfilling life is possible; preparation – the search for personal resources and external sources of help; rebuilding – taking positive steps towards meaningful goals; and growth – a sense of control over one’s life and looking forward to the future. In general terms, the transitions from one stage to the next are mediated by four processes – finding hope, taking responsibility, establishing a positive identity and finding meaning and purpose in life.

Where the rating scales for individual items refer to well-known programmes such as the Wellness Recovery Action Programme (WRAP), these are intended only as examples. Any similar programme would do. It is of course better to use a ‘manualised’ programme - a course of therapy that has been written in the form of a curriculum over a defined number of sessions, with learning goals for each session and pre-defined outcome measures. It is also best to use a treatment programme that has been validated, at least by change of outcome measures and preferably by demonstrating change in real-world outcomes such as reduced re-admission or re-offending. The use of a ‘manualised’ programme and appropriate training for the therapists ensures fidelity to the treatment programme as it was validated. However at present there is very little formal validation available for such programmes – this should be a topic for future development.

In general, if there is no problem of the sort referred to, a ‘0’ rating will apply e.g. for P2 Drugs and Alcohol where there is no history of any such problem.

Like the recovery items, those who are mostly rated ‘4’ are probably unlikely to be ready for a move to a medium secure setting, or to any setting at a lower level of security than their current placement; those mostly rated ‘3’ may be ready for a move from a high secure to a medium secure setting; those mostly rated ‘2’ may be ready for a move from medium to low security; those mostly rated ‘1’ may be ready for a move to an open or community placement – though the availability of a high level of community support, structure and supervision, mandated if necessary by legally binding conditional discharge with a power of recall, may be a part of such a decision. Finally, those rated ‘0’ in a range of areas relevant to their risk assessment may be ready for an absolute legal discharge though this should be an individualised decision in all cases.

|  | Cycle of change (Prochaska & DiClement 1983) | Engagement | Recovery (Andresen et al 2003) | Maslow (1943) |
| --- | --- | --- | --- | --- |
| O: ready for independence | Maintenance, stability | Taking personal responsibility | Growth | Self-actualisation |
| 1: ready for a move to supported community living e.g. conditional discharge or community treatment order | Maintenance, supported | Positive engagement | Rebuilding | Self-esteem, confidence, social standing |
| 2: ready for a move e.g. from medium to low security | Action / decisional balance | Active engagement | Preparation | Friendship and family relationships |
| 3: ready for a move e.g. from high to medium security | Contemplation & preparation, ambivalence | Passive engagement | Awareness | Safety and basics of life |
| 4: not ready to move down a level of security | Pre-contemplation. | Reluctance / resistance | Moratorium | Physiological needs |

**Programme Completion Item P1: Physical Health:**

This item rates the patient’s progress in actively managing their physical health. A preliminary step for most would be an education programme regarding physical health. This would be followed by a programme specifically focusing on physical health and recovery. While the scoring items refer to particular manualised programmes such as Solutions for Wellness, other programmes could as easily be substituted. For physical health, the emphasis has to be on having regular patterns of self-care including exercise, diet, sleep and engagement with clinics providing for any specific physical needs such as diabetes, cholesterol monitoring or other physical problems including regular health checks and national screening programmes

**Coding: P1. Physical Health**

| 0 | For a period of five years  Has taken responsibility for own active recovery and personal physical health,  A regular pattern of self-care, self-medication,  Self-presentation to primary care as appropriate. |
| --- | --- |
| 1 | Is self-medicating, self-caring and actively engaged with follow-up / maintenance programmes for physical health as appropriate e.g. self-monitors blood sugar if diabetic.  Has a regular dietary and exercise pattern and routine, has incorporated healthy eating and exercise programme into daily routine with minimum prompting.  Takes care of own appearance and health as a source of self-esteem and dignity. |
| 2 | As a minimum has successfully completed education programmes regarding physical health (e.g. ‘Solutions for Wellness’ or similar programme).  Evidence of change is sustained over time – at least twelve months e.g  Shows active interest in preparing healthy meals and takes exercise regularly, enjoys some form of sport or exercise.  May sometimes need prompting to adhere to physical health management programmes. |
| 3 | As a minimum has successfully completed a primary health care assessment and follow-up programme,  Takes a passive interest (contemplates, prepares for action) in balancing diet and exercise.  But only engages with healthy lifestyle options when prompted by staff to do so. |
| 4 | Has not yet successfully completed any programme concerning physical health awareness,  Does not choose healthy physical lifestyle options despite staff encouragement.  Is dependent on nursing care for many basic activities of daily living and self care. |

Information Quality: □ no information; □ staff observation; □ interview; □ family informants; □ clinical or police records (tick all informants that apply)

**“successfully completed” means has attended at least 90% of scheduled sessions in a programme during which the patient engaged fully, has actively participated and has shown the ability to personalise the content by giving examples from own experience relating to him/herself.**

**“Engaged” means enters into and commits to, as shown by consistency and initiative, effort and supportiveness of the goals of an activity or programme.**

**Programme Completion Item P2: Mental Health:**

This item rates the patient’s progress in actively managing their mental health. A preliminary step for most would be an education programme regarding physical and mental health. This would be followed by a programme specifically focusing on mental health and recovery. While the scoring items refer to particular manualised programmes such as Wellness Recovery Action Programme (WRAP), other programmes could as easily be substituted.

**Coding: P2. Mental Health**

| 0 | For a period of five years should have maintained an interest in active recovery and personal mental health, including  A regular pattern of self-care, self-medication,  Self-presentation to mental health team when necessary e.g. keeps appointments, recognises early signs of relapse and self-presents. |
| --- | --- |
| 1 | Should be self-medicating, self-caring and fully engaged with follow-up / maintenance programmes for mental health e.g. maintains contact with mentors and/or advocates as well as mental health professionals .  Has a regular pattern and routine of activities over the day, week and year.  Derives satisfaction from successful mental health achievements. |
| 2 | As a minimum has successfully completed a ‘Wellness’ education programme,  Evidence of change is sustained over time – at least twelve months e.g  Takes an active interest in balancing use of time between work (broadly defined), family and friends, leisure and creativity.  May need some prompting from staff and carers. |
| 3 | As a minimum has successfully completed a ‘wellness recovery action programme’ or equivalent.  Shows interest (contemplation / preparation) in learning about mental health and engages in programmes for relapse prevention. Needs encouragement.  May still need supervision of medication compliance. |
| 4 | Has not yet successfully completed any programme concerning illness awareness,  Requires supervised medication e.g. depot neuroleptic, observation swallowing meds, regular blood level checks. |

Information Quality: □ no information; □ staff observation; □ interview; □ family informants; □ clinical or police records (tick all informants that apply)

**“successfully completed” means has attended at least 90% of scheduled sessions in a programme during which the patient engaged fully, has actively participated and has shown the ability to personalise the content by giving examples from own experience relating to him/herself.**

**“Engaged” means enters into and commits to, as shown by consistency and initiative, effort and supportiveness of the goals of an activity or programme.**

**Programme Completion Item P3: Drugs and Alcohol:**

Because the clientele of a therapeutically secure mental health service is selected for severe mental illness, but the majority will have co-morbid substance misuse problems, and because the evidence that it is the combination of severe mental illness and intoxication that most predisposes to violence, the emphasis in forensic mental health services is on abstinence. The evidence for a sustained return to controlled drinking after a period of dependence is poor, and would not necessarily assist recovery from mental illness or reduce the risk of violence.

The aim is to participate fully in a graded series of programmes, starting with an education programme, progressing to an abstinence oriented recovery programme and followed by a maintenance / top-up programme.

**Coding: P3. Drugs and Alcohol**

| 0 | For a period of five years should have maintained an interest in active recovery with total abstinence for those with a history of substance misuse or dependence. |
| --- | --- |
| 1 | Should be fully engaged with follow-up / maintenance programmes as appropriate. Random screening is consistently negative.  Copes with ‘slips’ by seeking help.  Has regular patterns and routines in these domains.  Derives self-confidence from identity as an abstinent person in recovery. |
| 2 | As a minimum has successfully completed a full drugs and alcohol recovery programme.  Is working towards abstinence (action) e.g. by limiting/ending contact with former circle of users.  Evidence of change is sustained over time – at least twelve months e.g sustained abstinence.  May need continued prompting / guidance. |
| 3 | As a minimum has successfully completed an education programme regarding drugs and alcohol (if relevant).  Contemplation or ambivalence.. |
| 4 | Has not yet successfully completed any programme concerning substance misuse. Pre-contemplation. |

Information Quality: □ no information; □ staff observation; □ interview; □ family informants; □ clinical or police records (tick all informants that apply)

**“successfully completed” means has attended at least 90% of scheduled sessions in a programme during which the patient engaged fully, has actively participated and has shown the ability to personalise the content by giving examples from own experience relating to him/herself.**

**Programme Completion Item P4: Problem Behaviours:**

The expectation is that a preliminary stage would be fairly general consisting of enhanced thinking skills (ETS) and a selection of modules resembling dialectic behaviour therapy (a ‘balance’ programme). More specific programmes should follow, such as anger management (or CALM), healthy sexual functioning (or sex offender treatment programmes), victim impact and empathy programmes (including restorative programmes where possible) or full DBT programmes. Individual work should accompany such programmes. A primary goal should be to complete a Five WH programme (who, what, where, when, why) based on working through the book of evidence / witness statements presented at trial. Individual work may also include grief work, cognitive work for depression and cognitive work regarding the index offence or behaviour.

**Coding: P4. Problem Behaviours**

| 0 | For a period of five years should have had no offending behaviour or high risk behaviours for offending both specific to the patient and general.  Espouses pro-social beliefs, renounces pro-criminal beliefs. |
| --- | --- |
| 1 | Should be engaged with a well-balanced and regular daily and weekly programme of self-care, occupation and leisure.  Copes with behavioural ‘slips’ or new stresses by seeking appropriate help from the team in a timely way.  Derives confidence and self-esteem from changes associated with avoiding problem behaviours. |
| 2 | As a minimum has successfully completed offence related programmes e.g. anger management, healthy relationships and healthy sexual functioning, ‘5 Wh’ work, as individually appropriate.  Evidence of change is sustained over time – at least twelve months e.g. not requiring de-escalation. |
| 3 | As a minimum has successfully completed any general programmes regarding high risk behaviours such as meta-cognitive training enhanced thinking skills or ‘balance’ programme (DBT modules).  Patient accepts the need for change (contemplation/preparation) in psychological or interpersonal style specific to offending behaviour. |
| 4 | Has not yet successfully completed any programme concerning offence related behaviour.  Psychological / interpersonal aspects of offence related behaviour specific to the person are still in evidence  **OR** the patient is not yet contemplating change. |

Information Quality: □ no information; □ staff observation; □ interview; □ family informants; □ clinical or police records (tick all informants that apply)

**“successfully completed” means has attended at least 90% of scheduled sessions in a programme during which the patient engaged fully, has actively participated and has shown the ability to personalise the content by giving examples from own experience relating to him/herself.**

**“Engaged” means enters into and commits to, as shown by consistency and initiative, effort and supportiveness of the goals of an activity or programme.**

**Programme Completion Item P5: Self Care and Activities of Daily Living:**

,

The progression towards recovery here is likely to start with a basic course in kitchen hygiene and safety. An assessment such as the AMPS may underpin the programme that follows. Self-catering, including budgeting skills, shopping and use of public transport might usefully follow and give a purpose to the progressive use of leave as described in R4.

The aim is to achieve a well-balanced working week and a balanced life-style, in keeping with MOHO principles.

**Coding: P5. Self-Care and Activities of Daily Living**

| 0 | For a period of five years has maintained an interest in active recovery and personal mental and physical health, including  A regular pattern of self-care, occupation and leisure.  Has a network of informal as well as professional supports and carers. |
| --- | --- |
| 1 | Is self-caring and fully engaged with follow-up / maintenance programmes as appropriate.  Is engaged with a well-balanced daily and weekly programme of self-care, occupation and leisure.  Takes pride in / derives self-confidence from self-care and dignity. |
| 2 | As a minimum has successfully completed OT courses on self-catering, budgeting, shopping, use of public transport.  Evidence of change is sustained over time – at least twelve months e.g. Should be safe in workshops with shadow-boarded tools. Should be safe in kitchen-based groups.  May need prompting. |
| 3 | As a minimum has successfully completed assessments of abilities (AMPS, MOHO).  Shows a passive interest in aspects of self-care and ADL (contemplation – preparation). |
| 4 | Has not yet completed any programme concerning self-care or basic social skills, activities of daily living or interaction with others on the ward. Appears institutionalised / dependent over and above negative symptoms. |

Information Quality: □ no information; □ staff observation; □ interview; □ family informants; □ clinical or police records (tick all informants that apply)

**“successfully completed” means has attended at least 90% of scheduled sessions in a programme during which the patient engaged fully, has actively participated and has shown the ability to personalise the content by giving examples from own experience relating to him/herself.**

**“Engaged” means enters into and commits to, as shown by consistency and initiative, effort and supportiveness of the goals of an activity or programme.**

**Programme Completion Item P6: Education, Occupation and Creativity**

This refers to some of the elements regarded by Maslow as essential for self-actualisation. However aspects of these activities should be present for all. The progression here is from basic literacy, numeracy and communication skills to increasing engagement with occupational and leisure activities. Sport, awareness of current affairs and creative activities are considered broadly equivalent.

**Coding: P6 Education, Occupation, Creativity**

| 0 | For a period of five years has maintained a regular pattern of self-care, occupation and leisure.  Has a range of interests and activities including education, work (paid or un-paid), sport, creativity and awareness of current affairs |
| --- | --- |
| 1 | Should be engaged with a well-balanced regular daily and weekly programme of self-care, occupation and leisure. Some sport, creative or social / current affairs activities should be included.  Derives personal satisfaction from these activities and identifies with them. |
| 2 | As a minimum has successfully participated in programmes covering education and occupational skills and routines, and some creative activities (film club, creative writing, music, art, performance) or current affairs awareness.  Evidence of change/commitment to these activities is sustained over time – at least twelve months.  May need some prompting. |
| 3 | As a minimum has shown passive interest (contemplation – preparation) in any programme concerning literacy or study skills, occupations or creativity. |
| 4 | Has not yet engaged in any programme concerning literacy or study skills, occupations or creativity. May need direction or structuring to attend any such activities. |

Information Quality: □ no information; □ staff observation; □ interview; □ family informants; □ clinical or police records (tick all informants that apply)

**“successfully completed” means has attended at least 90% of scheduled sessions in a programme during which the patient engaged fully, has actively participated and has shown the ability to personalise the content by giving examples from own experience relating to him/herself.**

**“Engaged” means enters into and commits to, as shown by consistency and initiative, effort and supportiveness of the goals of an activity or programme.**

**Programme Completion Item P7: Family and Social Networks: Friendship and Intimacy**

The model here is a progression from quiet co-existence with fellow-patients through sustaining friendship without repetitive conflict to extending this style of relating to family and friends in the community. Formal family therapy may be an individualised part of this domain. However the successful management of relational therapeutic security, and in particular that aspect described as qualitative relational security emphasises the role of the nurses and other MDT members in recognising dysfunction in the ward based milieu of relationships and finding ways to address this.

**Coding: P7 Family and Social Networks, Friendship and Intimacy**

| 0 | For a period of five years has sustained good terms with all significant others, or else has found a safe way of getting on with them.  Is free of conflict with those in the immediate milieu (fellow patients / residents, formal and informal carers) and is capable of friendship (mutual support) with some.  Where dysfunction or conflict arises, this is not part of a pattern of repetition.  Where there are intimate relationships, these are consensual, and free of patterns of dysfunctional repetition. |
| --- | --- |
| 1 | Is be on good terms with all significant others, or else has found a safe way of getting on with them.  Is free of conflict with those in the immediate milieu (fellow patients / residents, formal and informal carers) and capable of friendship (mutual support) with some.  Where dysfunction or conflict arises, the person should be willing to seek help from the team in resolving this.  Where there is an intimate relationship or pattern of relationships, these are consensual and when dysfunction arises the person is /has been willing to seek help from the team in resolving this. |
| 2 | Family assessment/family therapy: As a minimum has freedom from conflict in family relationships (even if this includes the choice to minimise contact) and  Has mostly friendly interactions with those in the immediate millieu –  Is not prone to bullying, domination, exploitation or excessive isolation.  Evidence of change/commitment/consistency to these patterns of relating is sustained over time – at least twelve months. |
| 3 | As a minimum has a regular pattern of neutral or friendly interactions with staff and fellow-patients on neutral or shared topics of interest. |
| 4 | Has no interest in conversation or interaction with fellow patients, staff or with friends or relatives in the community OR  Has a pattern of dysfunctional or conflicting interactions and relationships. |

Information Quality: □ no information; □ staff observation; □ interview; □ family informants; □ clinical or police records (tick all informants that apply)

**“successfully completed” means has attended at least 90% of scheduled sessions in a programme during which the patient engaged fully, has actively participated and has shown the ability to personalise the content by giving examples from own experience relating to him/herself.**

**“Engaged” means enters into and commits to, as shown by consistency and initiative, effort and supportiveness of the goals of an activity or programme.**

**DUNDRUM-4: RECOVERY ITEMS**

This series of items is intended to provide a structured professional judgement instrument to assist the decision to move patients from higher to lower levels of therapeutic security. These should always be used in conjunction with the previous series of items concerning treatment programmes DUNDRUM-3. These items should be seen as qualitatively different from the DUNDRUM-1 triage security and DUNDRUM-2 triage urgency items. The coding has a parallel however. As before, this is a structured professional judgement tool. It is not intended that these items should absolutely determine the appropriateness or timeliness of a move from higher to lower levels of security or a delay in transfer. These items are intended only as a guide to what is relevant to the decision making process. These items should be regarded as ‘dynamic’ and should be reassessed at intervals, perhaps every three months or six months. These items may function in an analogous way to the dynamic ‘protective’ scales in the START (Webster et al 2009) and SAPROF (de Vogel et al 2009).

As before there may be legal or administrative barriers to the movement of patients from one level of therapeutic security to a lower level, based on need. These judicial / administrative factors are not included as an item here because the items listed are intended to inform the decision making process, including advice given to those with legal or administrative control over such moves – variously according to jurisdiction these decision makers may be Government Ministers or their advisers, Mental Health Review Boards or simply the clinical directors of secure and community mental health services. Because judicial / administrative factors are not included, the Recovery items may be used as an audit tool for the appropriateness and timeliness of such movements.

Those who are mostly rated ‘4’ are unlikely to be ready for a move from a high secure to a medium secure setting, or to any setting at a lower level of security than their current one; those mostly rated ‘3’ should be ready for a move from a high secure to a medium secure setting; those mostly rated ‘2’ should be ready for a move from medium to low security; those mostly rated ‘1’ may be ready for a move to an open or community placement – though the availability of a high level of community support, structure and supervision, mandated if necessary by legally binding conditional discharge with a power of recall, may be a part of such a decision. Finally, those rated ‘0’ may be ready for an absolute legal discharge though this should be an individualised decision in all cases.

We note that in a recent study, the HCR-20 dynamic items, the ‘C’ and ‘R’ sub-scales correlated with the levels of security to which patients had been allocated (Muller-Isberner, Webster & Gretenkord 2007).

**Recovery Item 1: Stability**

The decision to move a person from high to medium security, or from medium to low (minimum) security, or from low to community or open placements, and eventually to recommend an absolute discharge may be critically influenced by the extent to which the person has been stable and predictable over time.

‘Stability’ here is negated by evidence of relapse of positive symptoms, or evidence of violence or threatened violence to others rating above 4/6 on the DASA or requiring de-escalation, restraint, seclusion, additional medication or enhanced nursing observations.

**Coding: R1. Stability**

| 0 | Over a period of five years: no relapse or recurrence of problem behaviour, relapse unlikely; Advanced age may be taken into account |
| --- | --- |
| 1 | Relapses occur gradually over a period of weeks and in response to known patterns or precipitants. Signature signs and symptoms are known to carers and acknowledged by patient. Age may be taken into account. |
| 2 | Relapses may be abrupt, over days, but are predictable and patient has been stable for one year. Age may be taken into account. |
| 3 | Relapses may be abrupt and unpredictable, over days, but has been stable for one year. |
| 4 | Has no stable or predictable pattern of relapse of illness or recurrence of problem behaviours. |

Information Quality: □ no information; □ staff observation; □ interview; □ family informants; □ clinical or police records (tick all informants that apply)

**Recovery Item 2: Insight**

The most practical definition of insight is that given by Amador and David – dividing the concept into three independent elements – recognition of one’s own illness, recognition that one’s own symptoms such as delusions and hallucinations are the products of illness and acceptance of the benefits to one’s self of medication and other aspects of treatment.

The emphasis here is on appreciation that imparted information is relevant to the person himself or herself (note how the MacArthur structured professional judgement tools for assessing functional mental capacity divide this into understanding, reasoning and appreciation). Adherence or compliance is also relevant as evidence for the practical reliability of this quality.

Aspects of openness and trust are rated elsewhere.

**Coding: R2. Insight**

| 0 | Over a period of five years: in the event of relapse, actively seeks help; cooperates with crisis contingency plans; has previously cooperated with relapse contingency plans; acknowledges own need for professional help and more general supports in maintaining recovery. |
| --- | --- |
| 1 | Realistic appraisal of own risk of relapse; practical approach to relapse prevention; family and friends, if involved, are aware and supportive; has previously cooperated with relapse contingency plans when necessary. |
| 2 | Accepts own legal obligations and accepts treatment; is encouraged to do so by those friends or family who are most influential with him/her. |
| 3 | Acknowledges own legal obligations as a minimum. |
| 4 | Does not accept any aspect of own illness; does not accept legal obligations; does not engage actively in treatment or recovery oriented programmes. |

Information Quality: □ no information; □ staff observation; □ interview; □ family informants; □ clinical or police records (tick all informants that apply)

**Recovery Item 3: Therapeutic Rapport**

Working alliance and interpersonal trust are amongst the elements of therapeutic rapport. There is growing evidence that therapeutic rapport is one of the essential elements of meaningful outcome measurements for mental health. While this is commonly seen as a quality of the patient’s attitude to the professional carers, it has a reciprocal which is best described as the trust the professional carers feel for the patient. The patient’s sense of working alliance and interpersonal trust are aspects of an enduring disposition which non-the-less is amenable to change over the medium term.

**Coding: R3. Therapeutic Rapport**

| 0 | Over a period of five years: maintains contact regularly and spontaneously; is capable of transferring an open and communicative relationship from one professional to another at reasonable intervals. |
| --- | --- |
| 1 | Open and trusting with all members of multi-disciplinary team; capable of communicating matters relevant to risk; tolerates intrusion and restrictions on autonomy of treatment plan/ conditional discharge; not excessively dependent on particular individuals. |
| 2 | Capable of openness and trust with members of multi-disciplinary team; capable of limited exploration of current mental state as related to risk. |
| 3 | Tolerates daily intrusions and constrictions of therapeutic security; engages and participates in therapeutic and occupational programmes. |
| 4 | Does not tolerate monitoring or supervision – may seek to secrete, deceive or subvert. Negative disposition towards carers and professionals generally. |

Information Quality: □ no information; □ staff observation; □ interview; □ family informants; □ clinical or police records (tick all that informants apply)

**Recovery Item 4: Leave**

The graded use of leave outside the secure perimeter is an important guide to the readiness for progression from one level of therapeutic security to the next. Leave is an essential part of the rehabilitation process and it is necessary to take ‘therapeutic risks’ to ensure that institutionalisation does not occur, or to remedy early signs of institutionalisation. Institutionalisation should not be confused with the negative or deficit state of schizophrenia, which is characterised by lack of motivation, poverty of thought and affective flattening. Institutionalisation is characterised by dependence on the routines of the hospital ward, loss of skills in the activities of daily living such as doing one’s own laundry, shopping and cooking for oneself and others, tending to one’s own living space and property, and knowledge of the outside world generally e.g. using modern coinage, public transport, dealing with official forms and offices. While this item is not a rating of institutionalisation or of negative symptoms, this item is included because the necessity of taking therapeutic risks when assessing suitability for leave is so central to the process of rehabilitation and recovery in a forensic setting.

**Coding: R4. Leave**

| 0 | - For a period of at least five years has lived in the community and - has tolerated home visits and / or visits to place of work by members of the mental health team, both planned and unannounced. |
| --- | --- |
| 1 | Has used unaccompanied leave in the community for at least six months. |
| 2 | - Can use accompanied leave in the grounds of the medium secure hospital most of the time and - can use accompanied leave in the community with one member of staff - except when in relapse or when other indicators of risk are higher than usual. |
| 3 | - Can safely visit a medium secure setting prior to moving there from a high secure setting; - can use occasional leave to visit hospitals, family or other private venues - when accompanied by one member of staff. |
| 4 | - Represents such a high risk of absconding that can only leave a high secure setting under close the supervision of two or more member of staff. |

Information Quality: □ no information; □ staff observation; □ interview; □ family informants; □ clinical or police records (tick all informants that apply)

**Recovery Item 5: Dynamic Risk Items.**

Modern SPJ instruments such as the HCR-20 ‘Clinical’ or current items and the HCR-20 ‘Risk’ or future items are combined as ‘dynamic’ indicators of change over time. The S-RAMM, START and SAPROF may also describe these risk factors which are amenable to change. The HCR-20 ‘Risk’ or future items are usually rated for the eventuality of remaining in their present placement (‘in’) or moving to a less secure or open / community placement (‘out’). In general, if there is an obvious difference in the ratings for ‘in’ and ‘out’ then a move to a less secure place would increase the risk of violence.

As for Item T7, the rating for this item is not based on artificial actuarially calculated scores and probabilities. Instead the ratings are based on profiles of change over time.

**Coding: R5. Dynamic Risk Items**

| 0 | If the dynamic items have remained low and stable for a period of five years, and the Current / present items are similarly stable and low, the transition from conditional discharge in the community to absolute discharge may be considered. It may be that this can only safely be accomplished where there is consistent evidence of remission of symptoms (e.g. HCR-20 C3 =0 or Andreasen criteria for remission). |
| --- | --- |
| 1 | As for ‘2’, and - The dynamic scores should be equally low ‘in’ and ‘out’, while negative attitudes (HCR-20 C2) and impulsivity (C4) particularly would inhibit such a move. Active symptoms (C3), if they remain should be much reduced and stabilised. See R3 ‘Rapport’ regarding insight.(C1). Plans lack feasibility (R1) should be regarded as particularly important. |
| 2 | The move from medium to low therapeutic security may increase exposure to destabilisers (R2) and certain types of stress (R5), if so this should inhibit such a move while these issues are dealt with either through further psychological treatment, through addressing the choice of setting or level of support to be provided on moving etc; |
| 3 | - There is a score of ‘8’ or more on ‘C’ items, |
| 4 | - There is a score of ‘8’ or more on ‘R’ items for a move from present level of security to the proposed next lowest level, OR - There is a substantial difference (4 or more) between the ‘in’ and ‘out’ scores for ‘risk/future’ items (R1 to R5), when computed for any move to a lower level of security than the current placement. - C2 negative attitudes may also be particularly relevant here. |

Information Quality: □ no information; □ staff observation; □ interview; □ family informants; □ clinical or police records (tick all informants that apply)

**Recovery Item 6: Victim Sensitivity Items.**

This item presents special problems in balancing the rights and expectations of victims and patients. As a minimum, there should be a requirement that no fear or distress is afforded to the reasonable former victim or surviving relative of the victim. Some communities may be welcoming to the return of the patient, but some may not. If this were to engender a media campaign it would not be in the interests of the patient. An unsuccessful return to the former home community would have serious consequences for the future recovery of the patient. Accordingly, an essential part of the recovery process is the extent to which the needs of victims or their surviving relatives can be assessed and accommodated. This may be done by members of one of the other multi-disciplinary teams and/or a specialist victim support service making contact and offering information, support and advice, while avoiding breeching confidentiality. The needs of the victims can be incorporated into treatment and management plans, and conditions for leave and discharge. A continuing preoccupation with the former victim or with a predictable category of victim should also be rated here.

**Coding: R6. Victim Sensitivity Items**

| 0 | - Patient is capable of remorse for harm done to the victim and victim's relatives and - Victim or survivors have not been actively involved for 5 years (or are reconciled) and - Media interest has not been active for five years and patient has been living anonymously in the community and |
| --- | --- |
| 1 | - Patient accepts and complies with conditions regarding non-contact with victim or surviving relatives of victim or category of victims as appropriate or - Victim or survivors can be accommodated by reasonable conditions and restrictions on the movements of the patient and these have been observed by the patient while on leave from the hospital or - Victim or survivors would not be upset by patient being in community, includes geographic exclusions to prevent accidental meeting or - Media interest is no longer likely and patient should be able to live anonymously in the proposed community location for discharge.. |
| 2 | - Patient is capable of recognising the potential for hurt to the victim or category of victims. If at liberty would not represent a threat to them or - Victim or survivors can be accommodated by reasonable conditions and restrictions on the movements of the patient outside the hospital e.g. exclusion zones or - Victim would not be at risk of harm if patient was at liberty or - Media interest is no longer likely. |
| 3 | - Patient's preoccupation with specific victim or category of victims is encapsulated and no longer pervasive. - Victim or survivors are engaged in a process of liaison which respects confidentiality and the needs of both victim and patient, or - Victim or survivors would be upset / traumatised by contact but lesser harm original offence even if patient was in community or - Media interest is no longer active or intrusive but would still be hostile. |
| 4 | - Patient remains deluded or preoccupied with a former victim or category of victim and is still affectively motivated (e.g. angry, fearful) or - Victim or survivors remain actively engaged in petitioning against the movement of the patient or increase in access to the community or - Victim would be at risk of serious harm again if patient at liberty or - Media interest remains active, stigmatising and would pose a risk to the patient. |

Information Quality: □ no information; □ staff observation; □ interview; □ family informants; □ clinical or police records (tick all informants that apply)
